# Optic Neuritis Due to Lead Poisoning

**Published:** 1909-11-27

**Authors:** 


### Medicine.

### OPTIC NEURITIS DUE TO LEAD POISONING.

That optic neuritis often occurs without ascertainable cause is well enough known. In many such cases the patients seem to be perfectly sound except for their optic nerve inflammation, and the condition is referred to as a primary retro-bulbar optic neuritis. That at least some of these cases may be due to lead poisoning is brought home to us forcibly by papers published by Dr. J. Lockhart Gibson, of Brisbane, who has found that plumbic optic neuritis is of such frequency amongst Queensland children that if a child under eight years of age is brought to his consulting-room suffering from a recently-acquired internal squint, and this squint is found to be due to paralysis or paresis of one or both external recti, he almost invariably finds double optic neuritis, and upon further investigation discovers evidence or proof of plumbism. Unfortunately the ocular lesions are prone to occur without any wrist-drop or other sign of plumbism. The importance of this is obvious, seeing that non-removal of the cause may permit of the neuritis passing on to atrophy of the optic nerve, with more or less permanent blindness. The way in which the Queensland children get the lead into their systems is from their habit of holding on to the railings of verandahs and of gardens when they play. These railings are painted, and the paint, after a year or two of exposure to the weather, becomes powdery and easily detachable, owing to the formation of a soluble carbonate of lead. The latter adheres to the children's fingers, particularly in the hot weather; and it is noteworthy that many of the patients who have suffered most have been those who have been in the habit of biting their nails. The mode of ingestion of the lead in these cases is obvious. The symptoms vary in degree from mild to very severe. Dr. Gibson has described them in detail. The following three cases exemplify the mild, the medium, and the severe degrees respectively. There are, of course, both milder and severer cases than these.

### Some Illustrative Cases.

A child aged two years had defective vision, and presented a strabismus that had developed suddenly five days previously. The general health was good, though the child was a little fretful. There was paralysis of the right external rectus, and paresis of the left external rectus. Each optic disc exhibited several dioptres of swelling and well marked neuritis. There was a slight tendency to retraction of the head, but no vomiting, no convulsions, nor other evidence of meningitis or of intracranial tumour. There was no blue line upon the gums, but the urine contained 0.32 mg. of lead per litre. The result of treatment in this case was complete recovery.

A girl aged six years had been perfectly well until a fortnight previously, when she was seized with vomiting, violent abdominal pains, and constipation. Some days later her eyesight began to fail, and when seen in hospital she could not see at all. She was now not unlike a case of meningitis in that she was very restless and irritable, crying out as if in pain, with her neck held stiffly and her head slightly retracted. The pupils were sometimes large, sometimes small, and they reacted to light when they were large; but the eyeballs did not move on account of there being complete ophthalmoplegia externa. There was a well-marked condition of choked-disc and optic neuritis in each eye. There was complete insensibility to light, though the patient was not unconscious. Improvement fortunately set in rapidly, and by the twenty-fifth day after admission all the ocular muscles except the external recti were capable of at least some movement and the left eye could count fingers at a yard's distance. The optic neuritis had almost ceased at the end of a month, but it had been so extensive that it was followed by post-neuritic atrophy which kept the right eye almost blind; the left recovered sight to the extent of $\frac{6}{24}$.

A boy aged five years had been ill for two or three weeks with plumbic peripheral neuritis. The symptoms began with pain down the arms without paralysis; the pain was not constant, but came on in paroxysms. There was also much pain in the head and in the back of the neck, so that the lad cried out when either head or neck were moved. Added to this there was some retraction of the head, so that with optic neuritis as well it would have been very easy to make an erroneous diagnosis of tuberculous meningitis. The real cause of the mischief was determined by the discovery of lead in the urine. There was paralysis of both external rectus muscles, and the optic discs were swollen to the extent of about six dioptres. Fingers could be counted at eighteen inches. At a later date there was wrist drop, but this was not present to assist the diagnosis when the patient was most ill.

The result was one of permanent impairment of vision amounting almost to blindness from secondary optic atrophy; the paralyses got well, but four years after the acute illness the boy's eyesight was such that he could only count fingers at two feet with one eye, and at six inches with the other.

### Conclusions.

The absence of blue line upon the gums is an additional reason why the diagnosis of lead poisoning is apt to be particularly difficult in children. The blue line is due, of course, to the precipitation of the lead as black sulphide just beneath the surface of the mucosa; it is only when there is some degree of uncleanliness of the mouth that sulphuretted hydrogen is formed, especially close to the line where the gums adjoin the teeth. In the well-to-do who constantly use tooth-brushes, and in children whose teeth remain clean even when no tooth-brush is employed, it is the exception rather than the rule to find a blue line upon the gums even when there is a toxic amount of lead in the system. The value of testing the urine for lead as an assistance in the diagnosis of these cases is obvious.

